# A bioinformatic approach to identify pathogenic variants for Stevens-Johnson syndrome

**DOI:** 10.5808/gi.23010

**Published:** 2023-06-30

**Authors:** Muhammad Ma’ruf, Justitia Cahyani Fadli, Muhammad Reza Mahendra, Lalu Muhammad Irham, Nanik Sulistyani, Wirawan Adikusuma, Rockie Chong, Abdi Wira Septama

**Affiliations:** 1 Faculty of Pharmacy, Universitas Ahmad Dahlan, Yogyakarta 55164, Indonesia; 2 Research Centre for Pharmaceutical Ingredients and Traditional Medicine, National Research and Innovation Agency (BRIN), South Tangerang 15314, Indonesia; 3 Departement of Pharmacy, University of Muhammadiyah Mataram, Mataram 83127, Indonesia; 4 Center for Vaccine and Drugs, Research Organization for Health, National Research and Innovation Agency (BRIN), South Tangerang 15314, Indonesia; 5 Department of Chemistry and Biochemistry, University of California, Los Angeles, CA 90095, CA, USA

**Keywords:** bioinformatics, genetic variation, genomic, pathogenic variants, Stevens-Johnson syndrome

## Abstract

Stevens-Johnson syndrome (SJS) produces a severe hypersensitivity reaction caused by Herpes simplex virus or mycoplasma infection, vaccination, systemic disease, or other agents. Several studies have investigated the genetic susceptibility involved in SJS. To provide further genetic insights into the pathogenesis of SJS, this study prioritized high-impact, SJS-associated pathogenic variants through integrating bioinformatic and population genetic data. First, we identified SJS-associated single nucleotide polymorphisms from the genome-wide association studies catalog, followed by genome annotation with HaploReg and variant validation with Ensembl. Subsequently, expression quantitative trait locus analysis (eQTL) from GTEx identified human genetic variants with differential gene expression across human tissues. Our results indicate that two variants, namely rs2074494 and rs5010528, which are encoded by the *HLA-C* (human leukocyte antigen C) gene, were found to be differentially expressed in skin. The allele frequencies for rs2074494 and rs5010528 also appear to significantly differ across continents. We highlight the utility of these population-specific *HLA-C* genetic variants for genetic association studies, and aid in early prognosis and disease treatment of SJS.

## Introduction

Stevens-Johnson syndrome (SJS) and toxic epidermal necrolysis (TEN) are potentially life-threatening diseases [1]. In particular, SJS is a syndrome resulting from severe hypersensitivity reactions caused by infection with the herpes simplex or mycoplasma viruses, vaccinations, systemic diseases, certain agents, food, and drugs [2]. SJS occurs in the skin and mucous membranes in the orifices and eyes in mild to severe conditions, with abnormalities in the skin in the form of an erythema, vesicles, or bullae accompanied by purpura [3].

SJS/TEN is widespread in the mucocutaneous immune region, causing exfoliation of the skin on the mucosal surface [4]. The incidence of SJS, SJS/TEN, and TEN in the United States reported 9.2, 1.6, and 1.9 cases from 2009–2012 [5]. The incidence of SJS cases that occur in Indonesia is around 12 cases per year, with different causes [6]. The incidence in the United Kingdom from 1995–2013 there were 5.76 cases of SJS/TEN per million people per year [7]. In Korea, it is reported that the incidence rate of SJS/TEN from 2009–2013 was 3.96–5.03 and 0.94–1.45 per million people per year [8]. Events caused by SJS, SJS-TEN, and TEN have an average of 5.3, 0.8, and 0.4 cases per million children each year [9]. The mortality rate of 4.8-9% in SJS, 19.4–29% in the SJS/TEN case, and 14.8–48% in TEN [10]. SJS can appear with non-specific fever symptoms that cause malaise, headache, cough, and rhinorrhea. On the skin, patients suffering from SJS can have polymorphic lesions and mucous membranes with marked skin blisters and erosion [11].

Therefore, primary prevention is the best mitigation for SJS. SJS is categorized as a severe cutaneous adverse reaction (SCAR), and several drugs have been implicated in disease pathogenesis. Non-steroidal anti-inflammatory drugs and other multi-ingredient formulations are widely used to relieve the symptoms. Several studies are reporting adverse skin drug reactions that are often SJS-associated with severe ocular complications [12]. Prevention is possible if patients who are susceptible to this SCAR when prescribed certain drugs are identified. Besides, the genomic variants are known to have an important role in SJS progression. However, little information revealed the specific variant as a biological risk gene in SJS. A previous study revealed associated variants in SJS progression (rs2844665, rs3815087, rs3130931, rs3130501, rs3094188, and rs9469003). In allopurinol-induced SJS and TEN, rs9469003 can detect an effect similar to that seen with an allele frequency risk of 15% and an associated risk of 99% [13]. Even though several studies have been exploited previously, a limited number of variants summarized the variants associated with expression in tissue-related SJS. This study aims to investigate the variants associated with SJS through a bioinformatic-based approach and further prioritize the biological risk variants. Besides, the pattern of gene expression profiles and population allele frequencies of genetic variants were assessed using various databases. Here, The results will enable future studies to assess whether these variants may be associated with various infectious risks for SJS/TEN, as well as SJS progression and disease susceptibility.

## Methods

**Fig. 1.**
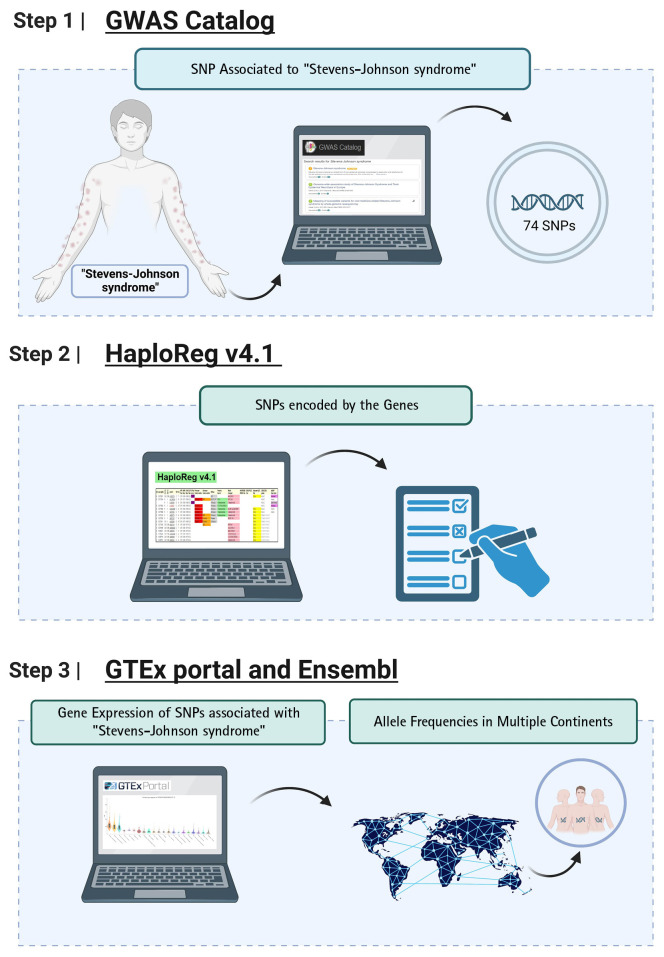
Bioinformatics workflow for the identification of genetic variants associated with Stevens-Johnson syndrome. GWAS, genome-wide association studies; SNP, single nucleotide polymorphism; *HLA-C*, human leukocyte antigen C.

Genomic information not only can be leveraged to identify the variant-associated disease, but it can also be translated into actionable knowledge for the disease. SJS is one of the severe skin reaction due to genomic risk factors. In this study, we used a bioinformatic-based approach to prioritize the pathogenic variants that potentially trigger the SJS. Detailed information regarding the study design has been depicted in Fig. 1. We used the keyword "Stevens-Johnson syndrome (SJS)" to derive SJS associated from the genome-wide association studies (GWAS) National Human Genome Research Institute (NHGRI) Catalog database (http://www.ebi.ac.uk/gwas accessed December 19, 2022). SJS associated with 74 single nucleotide polymorphisms (SNPs) were obtained and further analysis was carried out using HaploReg (version 4.1). Further analysis yielded a total of 41 SNPs with a significance value of p-value < 10^-8^. This value is used to account for several tests in the GWAS catalog. These values are widely used to identify associations between variants and shared genetic traits with adjacent gene expression [14]. Furthermore, an evaluation was carried out between the relationships of various genetic variants and gene expression profiles using expression quantitative trait locus (eQTL) using the GTEx Portal database (http://www.gtexportal.org/home/ accessed December 19, 2022), which was found by gene expression from various networks. The *HLA-C* (human leukocyte antigen C) genetic variant is present in human skin and tissue (lower extremities) exposed to sunlight obtained from the GTEx Portal database. Then confirm the variant using the Ensembl Genome Browser (https://www.ensembl.org/index.html accessed December 19, 2022). Furthermore, the allele frequencies of variants associated SJS were evaluated in different populations including African, American, East Asian, European, and Southeast Asian populations. Samples from each region consisted of 331 individuals (Africa), 199 individuals (America), 247 individuals (East Asia), and 136 individuals (Southeast Asia). Then, to find out the function of the various gene variants, an evaluation was carried out using the SNP Nexus database (https://www.snp-nexus.org accessed December 19, 2022).

## Results and Discussion

### Identification of genomic variants for SJS

We first identified SNPs associated with SJS from the GWAS database, resulting in 74 SNPs associated with SJS. We identified 41 unique SNPs associated with SJS after removing all SNPs duplication (Table 1). Based on the number of SNPs obtained, candidate SNPs were further constrained and prioritized using HaploReg version 4.1, with a p-value of <10^-8^. Based on the findings presented in Table 2, we focused on two genomic variants from the same gene that qualify as the biological risk SNPs for SJS from this study.

Through our integrative bioinformatics approach, two variants with a missense mutation (rs2074494, rs5010528) that encoded the *HLA-C* genes were prioritized as the biological risk SNPs for SJS. SJS is characterized by necrosis and shedding of the epidermis, known as the triad of disorders of the vesiculobullous skin, orifice mucosa, and eyes, accompanied by severe general symptoms [6]. It was also reported that the *HLA-C* gene has an important role in protecting against cancer and viruses. However, the *HLA-C* gene may also be involved in allograft rejection, the state of preeclampsia, and is also present in autoimmune diseases [15]. The diagnosis of SJS/TEN is a blistering autoimmune disease. which is included in linear IgA dermatosis and paraneoplastic pemphigus but is also present in pemphigus vulgaris and bullous pemphigoid, acute generalized exanthematous pustulosis, and later disseminated drug persistently erupting bullosa and staphylococcal scalded skin syndrome [16].

### *HLA-C* gene expression across 16 human tissues

**Fig. 2.**
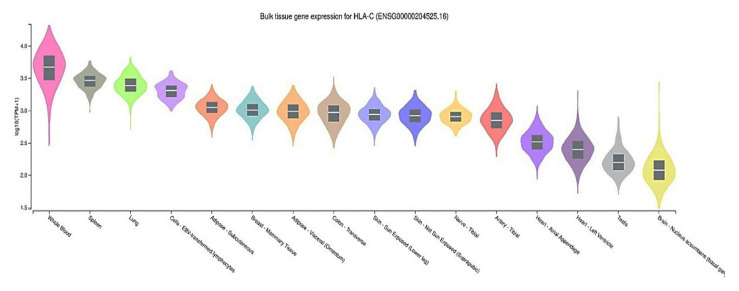
Human leukocyte antigen C (*HLA-C*) gene expression–associated with Stevens-Johnson syndrome in several human tissues from the GTEx Portal.

To evaluate *HLA-C* gene expression in human tissues, we used the GTEx Portal database (http://www.gtexportal.org/), which contains gene expression levels in various tissues. eQTL annotation comprises the most apparent functional consequences of genetic variation. Whole blood, spleen, lung, and lymphocyte cells demonstrate the highest *HLA-C* gene expression across the 16 human tissues analyzed from GTEx in Fig. 2. Furthermore, we have found that the ID SNPs rs2074494 and rs5010528 have similar gene expression variation in the Sun-Exposed Skin (Lower Leg). SJS is acute and can cause death occurs, therefore this disease is an emergency disease on the skin [16]. One Sun-Exposed Skin that is often exposed is exposure to ultraviolet radiation (UVR). In general, exposure to UVR has been reported as a risk factor for SJS and TEN [17].

### *HLA-C* gene expression in the sun-exposed skin

Human leukocyte antigen (HLA) class I genes, including *HLA-A*, *HLA-B*, and *HLA-C*, have been reported as the loci most strongly associated with susceptibility to all types of SJS and TEN, including cold medicine-related (CM-SJS) and TEN with severe ocular complications (SOC). Although non-synonymous substitutions affecting peptide binding or *HLA* molecular conformation have been considered significant factors in the pathogenesis of immunological diseases, indeed, different *HLA-C* expression levels have been reported for the different alleles, with higher HLA-C expression leading to increased Tc (*Trypanosoma cruzi*) responses and adverse effects in Crohn's disease. It was also reported that genetic variation in *HLA-A* and other autosomal genes has been identified as a risk factor for SJS/TEN associated with flu drugs with SOC such as CM-SJS or TEN with SOC [18].

When individuals with a genetic background containing SJS/TEN with SOC susceptibility factors are infected by some viral or microbial infection, they develop abnormal immune responses [19]. It is reasonable to presume that there is an interaction between HLA multiplication and susceptibility genes such as *HLA-A* and *TLR3* [20], *HLA-A* and *REC14-32*, and *HLA-A* and *PTGER3* [21]. Several susceptibility genes for SJS/TEN CM associated with SOC may be involved in the formation of functional networks. An imbalance in this gene can trigger the mucocutaneous inflammation seen in patients with SJS/TEN associated with CM with SOC. SJS/TEN with SOC in the acute stage shows inflammation of the skin and ocular surface and oral mucosal erosions and paronychia [12].

### Relationship between *HLA-C* gene and eQTLs from the GTEx database

Gene expression of SJS in human tissue was evaluated via the GTEx Portal database. This aims to determine the gene expression level in various tissues including in the skin tissue. We identified genomic variations from *HLA-C* gene expression using the GWAS catalog database and found 74 SNPs. From these analyses, we determined top 10 SNPs with the highest p-values. We were further processed with the variant annotation tool SNPnexus to determine the annotation of prioritized SNP variations. After these analyses, two statistically significant SNPs were obtained and prioritized. In this case, we prioritized 2 SNPs at risk for SJS based on an analysis of the number of SNPs expanded using HaploReg version 4.1 and a p-value of < 10^-8^ to determine the functional annotation of the SNPs. The results of genetic variation are shown in Table 3.

**Fig. 3.**
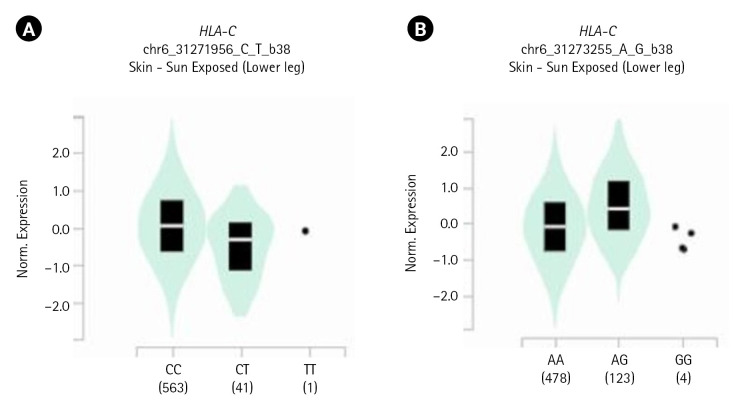
Human leukocyte antigen C (*HLA-C*) gene expression for each genotype of the single nucleotide polymorphisms: (A) rs2074494 and (B) rs5010528.

Table 3 shows the two identified variants (rs2074494 and rs5010528) encoded the *HLA-C* gene in differential tissue expression in the human skin. By using the GTEx portal (http://www.gtexportal.org/home/), we further emphasized that the variants of rs2074494 and rs5010528 encoded the *HLA-C* genes were a higher expression in the skin tissue to Table 3 and Fig. 3.

### Allele frequencies of SJS candidate variants across continents

**Fig. 4.**
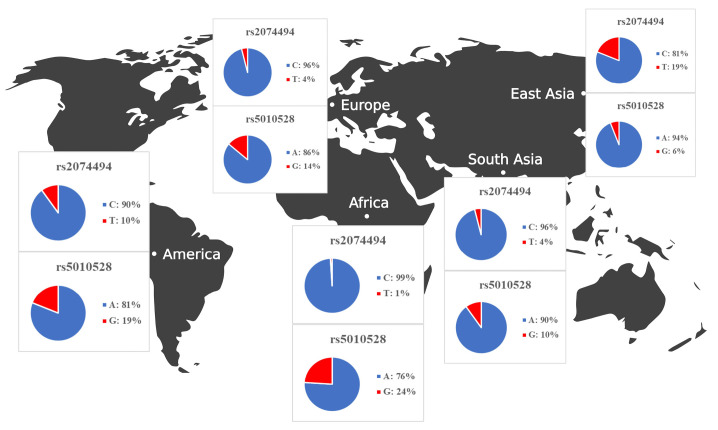
Summary of allele frequency analysis on human leukocyte antigen C (*HLA-C*) gene expression in Africa, America, East Asia, Europe, and Southeast Asia.

Once we identified the candidate *HLA-C* expression–associated variants, we set out to determine the allele frequencies across transcontinental populations as shown in Table 4. The allele frequencies for both variants were evaluated in different population including African, American, East Asian, European, and Southeast Asian populations. Samples from each region consisted of 331 individuals (Africa), 199 individuals (America), 247 individuals (East Asia), and 136 individuals (Southeast Asia). We extracted the allele frequencies in Africa, America, East Asia, Europe, and Southeast Asia from the Ensemble Genome Browser (http://www.ensembl.org). Allele frequencies across populations differ for each *HLA-C* variant. Table 4 and Fig. 4 show gene expression levels at higher frequencies of the rs5010528 associated (G) allele and rs2074494 associated (T) allele. At population frequencies of the rs5010528 (G) allele, the Asian population (East Asia and Southeast Asia) is expressed at a much lower level than that from the populations of Africa, America, and Europe.

The allele frequency of the “rs2074494” T allele in the African population is expressed at a much lower level than that of the populations of America, Europe, and Southeast Asia. Taken together, the allele frequencies of the variants “rs2074494” and “rs5010528” indicated the contribution of differential variant prevalence for *HLA-C* gene expression.

Another study revealed that *HLA-S* gene which were reported to be potential associated in chickenpox disease [22]. In this study, we investigated the skin tissue expression of the *HLA-C* gene, which has been linked to SJS and can lead to SCAR infection. Notably, the variants associated with *HLA-C* expression have not been reported for SJS. Basic research on the genetics of SJS and TEN to date has focused on HLA, the system associated with the presence of specific receptors, cytotoxic proteins, and the part of immunocytes during disease pathogenesis [23]. Considering the global impact of SJS, examining the distribution of *HLA-C* variants may be an essential quest that allows further understanding of global disease susceptibility.

The *HLA* gene encodes several molecules that are crucial to the immune system. With that in mind, a strong relationship between *HLA* genes and autoimmune diseases has been demonstrated for more than half a century [24]. Findings that most patients with carbamazepine-induced SJS and CBZ-SJS/TEN toxic epidermal necrolyses have an associated HLA-B*15:02 in an Asian population. In contrast, the association with HLA-A*31:01 was only reported in Japan and Europe. and has a novel association between HLA-A*31:01 and CBZ-SJS/TEN in Indians [25]. The association with various *HLA* genes can then be analyzed using publicly available databases. We used publicly available databases like the GTEx Portal, SNPnexus, and Ensembl. We identified genetic variants associated with *HLA-C* expression in skin tissue, the leading site of SCAR infection in SJS disease. It has previously been reported that specific *HLA* genotypes have been associated with the occurrence of severe skin disease due to drug-induced side effects (SCARs), which cases are included in SJS/TEN [26].

The allele frequencies in all populations differ for each SNP, as shown in Fig. 4. In general, it is known that the G and A allele frequencies for rs2074494 and rs5010528 were also seen to have a lower frequency in Southeast Asia (rs2074494, 4% and rs5010528, 14%), Europe (rs2074494, 4% and rs5010528, 16%), and East Asia (rs2074494, 19% and rs5010528, 6%), compared with American (rs2074494, 10% and rs5010528, 24%) and African (rs5010528, 24%) populations. In conclusion, by leveraging a bioinformatic-based approach it is revealed the pathogenic variants that are potentially associated with SJS. We propose that these variants could be used for further study to identify the SJS diagnostic biomarker as well as for prognosis. However, we acknowledged that there are limitations to the bioinformatic-based approach used to investigate the genetic variants associated with SJS. One of the main limitations is that not all the variants necessarily have genes that encode them (i.e., non-coding variants), and even if they do, these genes or genetic variants may not be suitable drug targets. Nonetheless, clinical validation is recommended as a next step to confirm our findings and gain a better understanding of the underlying etiology and functional effect of the SJS disease.

### Conclusion

In this study, we conducted a comprehensive bioinformatic analysis of SJS from genomic databases, revealing differential tissue expression of the *HLA-C* gene across 16 human tissues. Even though *HLA-C* is highly expressed in whole blood, spleen, lung, and lymphocyte cells, the relevant disease variants (rs2074494 and rs5010528) are differentially expressed in the skin tissue. Overall, alleles for rs2074494 and rs5010528 have lower frequencies in Southeast Asia (rs2074494, 4% and rs5010528, 14%), Europe (rs2074494, 4% and rs5010528, 16%), and East Asia (rs2074494, 19% and rs5010528, 6%), as compared to the American population (rs2074494, 10% and rs5010528, 24%) and the African (rs5010528, 24%) population.

## Figures and Tables

**Table 1.** SNPs from the GWAS catalog with a significance of p-value < 10^-8^

Variant and risk allele	p-value
rs6457109	3 × 10^-16^
rs7760545	4 × 10^-16^
rs35835721	8 × 10^-15^
rs137899365	3 × 10^-14^
rs60581484	2 × 10^-13^
rs1131151	4 × 10^-12^
rs2074494	5 × 10^-12^
rs4917014	8 × 10^-11^
rs1562468327	6 × 10^-10^
rs199755581	7 × 10^-10^
rs11509487	1 × 10^-9^
rs9469003	2 × 10^-9^
rs4471527	2 × 10^-9^
rs28381346	5 × 10^-9^
rs6500265	6 × 10^-9^
rs199755581	6 × 10^-9^
rs114908185	7 × 10^-9^
rs1297852527	9 × 10^-9^
rs1371146120	1 × 10^-8^
rs77491650	1 × 10^-8^
rs4471527	1 × 10^-8^
rs77542827	1 × 10^-8^
rs3130501	2 × 10^-8^
rs536142737	2 × 10^-8^
rs16957893	2 × 10^-8^
rs2734583	2 × 10^-8^
rs150289893	2 × 10^-8^
rs1286845082	2 × 10^-8^
rs548089948	2 × 10^-8^
rs3094188	3 × 10^-8^
rs55765602	3 × 10^-8^
rs77542827	3 × 10^-8^
rs778096762	3 × 10^-8^
rs1597607761	3 × 10^-8^
rs1391213386	3 × 10^-8^
rs374138762	4 × 10^-8^
rs879656274	4 × 10^-8^
rs1211926109	4 × 10^-8^
rs116953913	5 × 10^-8^
rs1263106470	6 × 10^-8^
rs5010528	8 × 10^-8^
	

SNP, single nucleotide polymorphism; GWAS, genome-wide association studies.

**Table 2.** Stevens-Johnson syndrome variant and risk allele that codes for prioritized SNPs

Variant and risk allele	Variants near risk allele (r^2^ > 0.8)	p-value	Gencode	Allele type
rs2074494	rs1050276	5 × 10^-12^	*HLA-C*	Missense
rs5010528	rs1050409	8 × 10^-8^	*HLA-C*	Missense

SNP, single nucleotide polymorphism.

**Table 3.** *HLA-C* eQTL analysis from the GTEx Portal database

SNP ID	Gencode ID (ENSG00000-)	Gene symbol	p-value	Effect size	Tissue	Expression level
rs2074494	204525.16	*HLA-C*	7.2 × 10^-10^	–0.48	Artery-Tibial	CC>CT>TT
	204525.16	*HLA-C*	13 × 10^-6^	–0.50	Muscle-Skeletal	CC>CT>TT
	204525.16	*HLA-C*	15 × 10^-6^	–0.78	Brain-Nucleus accumbens (basal ganglia)	CC>CT>TT
	204525.16	*HLA-C*	17 × 10^-6^	–0.51	Heart-Left ventricle	CC>CT>TT
	204525.16	*HLA-C*	46 × 10^-6^	–0.58	Testis	CC>CT>TT
	204525.16	*HLA-C*	36 × 10^-5^	–0.39	Skin-Sun exposed (lower leg)	CC>CT>TT
rs5010528	204525.16	*HLA-C*	5.4 × 10^-26^	0.56	Adipose-Subcutaneous	AA>AG>GG
	204525.16	*HLA-C*	1 × 10^-21^	0.41	Whole blood	AA>AG>GG
	204525.16	*HLA-C*	1.4 × 10^-14^	0.51	Adipose-Visceral	AA>AG>GG
	204525.16	*HLA-C*	4.1 × 10^-14^	0.49	Lung	AA>AG>GG
	204525.16	*HLA-C*	1.1 × 10^-10^	0.68	Spleen	AA>AG>GG
	204525.16	*HLA-C*	2.1 × 10^-9^	0.51	Heart-Atrial appendage	AA>AG>GG
	204525.16	*HLA-C*	5.7 × 10^-8^	0.28	Colon-Transverse	AA>AG>GG
	204525.16	*HLA-C*	9.9 × 10^-8^	0.38	Skin-Not sun exposed (suprapublic)	AA>AG>GG
	204525.16	*HLA-C*	5.1 × 10^-7^	0.72	Cells-EBV-transformed lymphocytes	AA>AG>GG
	204525.16	*HLA-C*	7.0 × 10^-7^	-0.25	Nerve-Tibial	AA>AG>GG
	204525.16	*HLA-C*	7.7 × 10^-7^	0.31	Nerve-Tibial	AA>AG>GG
	204525.16	*HLA-C*	1.6 × 10^-6^	0.34	Breast-Mammary tissue	AA>AG>GG
	204525.16	*HLA-C*	1,8 × 10^-6^	0.32	Skin-Sun exposed (lower leg)	AA>AG>GG

Source: Expression Quantitative Trait Loci (eQTL) obtained from the GTEx Portal. *HLA-C*, human leukocyte antigen C; eQTL, expression quantitative trait loci; SNP, single nucleotide polymorphism.

**Table 4.** Analysis of allele frequencies for the *HLA-C* gene from SNPnexus variant annotation.

SNP ID	Position (hg38)	Gene symbol	Location	Allele		Allele frequency (n)				
				Ref	Alt	AFR	AMR	EAS	EUR	SAS
rs2074494	Chr6:31271956	*HLA-C*	Missense	C	T	T: 0.008 (10)	T: 0.099 (69)	T: 0.189 (191)	T: 0.037 (37)	T: 0.040 (39)
rs5010528	Chr6:31273255	*HLA-C*	Missense	A	G	G: 0.243 (321)	G: 0.187 (130)	G: 0.056 (56)	G: 0.138 (139)	G: 0.099 (97)

*HLA-C*, human leukocyte antigen C; SNP, single nucleotide polymorphism; Ref, Reference; Alt, slternate; AFR, Africa; AMR, America; EAS, East Asia; EUR, Europe; SAS, Southeast Asia.
